# Development of Chinese-Style Sausage Enriched with Djulis (*Chenopodium formosanum* Koidz) Using Taguchi Method: Applying Modern Optimization to Indigenous People’s Traditional Food

**DOI:** 10.3390/foods13010091

**Published:** 2023-12-26

**Authors:** Pei-Ling Chung, Ku-Sang Lufaniyao, Mohsen Gavahian

**Affiliations:** 1 Department of Pet Care and Grooming, Tajen University, Pingtung 90741, Taiwan; plchung@tajen.edu.tw (P.-L.C.); ssw591208@gmail.com (K.-S.L.); 2 Department of Food Science, National Pingtung University of Science and Technology, Pingtung 91201, Taiwan

**Keywords:** pork sausage, red quinoa, Taguchi method, optimization, indigenous foods, food nutrition improvement, food security

## Abstract

Djulis (Taiwanese quinoa) has gained popularity among researchers due to its biological characteristics and rich nutritional value. Incorporating Djulis is expected to enhance the sausage’s texture, flavor, and storage stability due to the presence of antioxidants and nutritional components. However, limited studies focus on product development based on this emerging health-promoting ingredient in the food industry. This study aims to develop Chinese-style sausage enriched with Djulis using the Taguchi L_9_(3^4^) orthogonal matrix methodology and evaluate the influence of four factors, including un-hulled to hulled Djulis ratios of 0, 50, 100% (A), backfat-to-lean meat ratios of 0/100, 30/70, 50/50% (B), cooking temperature of 55, 75, 95 °C (C), and nitrite content of 0.03, 0.05, 0.07 g/kg (D) on products’ sensory and physicochemical properties. The optimal Taguchi formulation was then verified and compared with conventionally formulated sausage (original sausage) in terms of hardness, springiness, gumminess, CIE color values, and peroxide value (POV). The optimal formulation was A_3_B_2_C_2_D_3_, which consisted of 16.8% hulled Djulis, 30/70 backfat-to-lean meat ratio, 75 °C cooking temperature, and 0.03 g/kg nitrite content. The most influential independent parameters were identified as B > A > C > D, placing Djulis incorporation as the first runner-up, just after the backfat-to-lean meat ratio. Optimized condition verification identified the Signal-to-Noise ratio (S/N) of 16.63. Comparing the optimized Djulis-enriched sample and the original sausage indicated similar CIE L*, a*, b*, hardness, and springiness but different gumminess according to Texture Profile Analysis (TPA). The Djulis-enriched sausage at the optimized formulation had a significantly lower POV compared to the control sample (4.65 vs. 9.64 meq/kg), which was found to be correlated with Djulis antioxidant effects with 2,2-diphenyl-1-picryl-hydrazyl-hydrate (DPPH) free radical antioxidant activity of 62.37%. This suggests that Djulis effectively mitigates sausage organoleptic deterioration. Djulis sausage, with natural antioxidants and reduced fat content, could cater to consumer preferences and enhance the market for the food industry and indigenous farmers.

## 1. Introduction

Chinese-style sausage (Xiang Chang) is a traditional meat product in Taiwan with a peculiar flavor and diversity. This sausage consists mainly of pork (including fatty and lean meat), sausage casings, sugar, salt, five-spice powder, white pepper, and other ingredients. Different flavors are achieved by seasoning with various additional ingredients. A report on the global sausage and hot dog market published in 2016 indicated that in 2014, the Asia-Pacific region accounted for 25.81% of the worldwide sausage and hot dog market revenue. The market size was projected to increase from $64.7 billion in 2014 to $80.4 billion in 2021 [1]. According to Nielsen Corporation statistics, Black Bridge, the largest processed meat brand in Taiwan, has an average market share of approximately 35% for Chinese-style sausages, ham, bacon, and Vienna sausages sold in refrigerated counters. The market share for individual Chinese-style sausage products alone reaches 60%. Based on the brand’s annual revenue of around 42 million US dollars, the sausage market in Taiwan alone exceeds 77 million USD [2].

Djulis (red quinoa), a native crop to Taiwan, has attracted much attention because of its rich nutritional value and high content of essential amino acids, minerals, and trace elements, with several health benefits and therapeutic effects [3,4,5]. Moreover, Djulis also exhibits excellent antioxidant properties [3,6]. Enriching meat products with such health-promoting ingredients can increase farmers’ income and food manufacturers’ competitiveness, providing consumers with healthier choices. Using the traditional ingredient of indigenous people, i.e., Djulis, could bring food back to its roots. Moreover, some consumers are concerned about processed meats (e.g., sausages), mainly due to the potential presence of N-nitroso compounds [7], chemical compounds associated with serious health problems [8]. It has been reported that fortifying processed meat with some plant-based compounds, such as inulin, could attenuate the formation of nitroso compounds [9]. Accordingly, formulating meat products with bioactive-rich plant material (e.g., Djulis) and optimizing the formulation based on nitrite content can potentially reduce such consumers’ concerns. Djulis contains 14% protein (~double that of rice), 14% dietary fiber (~seven times more than sweet potatoes), and abundant calcium, iron, zinc, and selenium. It also contains essential amino acids, such as isoleucine, lysine, and histidine. Djulis seeds harbor high levels of antioxidant enzymes such as peroxidase, catalase, and superoxide dismutase, along with compounds such as saponins, betalains, flavonoids, polyphenols (e.g., rutin), chlorogenic acid, catechin, and antioxidants. These could offer health-promoting effects on the human body. Djulis-based product categories mainly include general food and beverages, topical products, and healthy foods. So far, more than 200 Djulis-based products have been introduced to the market, most of these products were added in the recent years. With the rise of consumers’ health awareness, Djulis-related products are also gradually increasing their market competitiveness [10]. Studies indicate that oral administration of Djulis extracts effectively, such as lowering blood pressure, and preventing coronary atherosclerosis [10,11,12]. Further, in a 2023 study, it was proven to have the potential to be a healthy food that protects the stomach [13].

Djulis-enriched products have been used in staple foods such as bread as well as mixed grains, noodles, convenience foods (e.g., salads and sandwiches). It can be seen that “food and beverages” are still the mainstream of current Djulis products. Overall, the Asia-Pacific market continues to pay attention to emerging products such as Djulis. Also, Djulis is added to foods as a nutritional supplement. In addition, a new trend of making Djulis into meal replacement drinks has emerged [14]. However, information on its incorporation into sausage formulation is limited.

Previous studies have extensively explored the addition of various grain products to sausages. For example, adding sorghum distillers’ grains can replace starch as an ingredient in sausage production, improving appearance, texture, and mouthfeel [15].

At the same time, developing a new product could be challenging unless advanced optimization approaches considering multiple variables can be utilized. Taguchi is an optimization approach to improving the quality of products, reducing costs, and providing robust design solutions. It has gained popularity in the food industry to optimize the food process [16] and formulation [14]. This includes evaluating the production of healthy foods such as Ganoderma pellets and even the foam ratios of food models using the Taguchi method [17]. Some researchers preferred using other methodologies instead of Taguchi, as illustrated in Table 1. The reasons behind this, in some cases, are its provision of relative results, necessitating more precision in indicating the parameter with the highest effect on the performance characteristic value. Also, due to orthogonal arrays not testing all variable combinations, the method is unsuitable when all relationships between variables are essential. Moreover, the Taguchi method has been criticized in the literature for its difficulties in accounting for interactions between parameters. Furthermore, the Taguchi methods are offline and, therefore, unsuitable for a dynamically changing process such as a simulation study. Additionally, the Taguchi method focuses on designing quality rather than correcting for poor quality; they are most effective at the early stages of process development. After design variables are specified, experimental design may be less cost-effective. Despite the limitations, the numerous benefits of the Taguchi method make it a promising technique for process design, new product development, and optimization in the food industry. Its benefits include prioritizing a mean performance characteristic value close to the target value over certain specification limits, thus improving the overall product quality, being straightforward to apply to many engineering situations, making it a powerful yet simple tool to narrow down the scope of a research project or to identify problems in a manufacturing process. It also allows for the analysis of many different parameters with a reduced number of experiments. For example, a process with eight variables, each with three states, would require 6561 (_3_^8^) experiments to test all variables, but Taguchi’s orthogonal arrays require only 18 experiments. This enables the identification of key parameters influencing the performance characteristic value, focusing further experimentation on impactful variables while disregarding those with minimal effects.

According to the above, the present research aims to develop a potentially healthier sausage by adding Djulis to Chinese-style sausage, evaluating the effects of formulation on the product’s quality parameters, optimizing the formulation by the Taguchi method, verifying the optimized formulation, and assess its physicochemical properties and consumer’s acceptability.

**Table 1:** Some of the advantages and limitations of the Taguchi method in comparison with other alternative methodologies.

	Taguchi Method	Other Methods, such as Full Factorial	Reference
Advantages *	Reduced number of experiments.	Higher accuracy.	[18,19,20]
	2. Reduced costs and time.		
Limitations	The method could be less accurate than the full factorial method.	Large number of experiments.	[20,21]
		2. Higher costs and more time required to perform the experiments.	

* The information in this Table is provided as examples only, and the advantages and limitations of this method may vary case by case.

## 2. Materials and Methods

### 2.1. Materials

The un-hulled (whole grain) and hulled Djulis (*Chenopodium formosanum*) were obtained from a farm in Majia Township, Pingtung, Taiwan. Boston butt pork and pork backfat were bought from a local chain supermarket (Carrefour Co. Ltd., Pingtung, Taiwan). Hog casings were purchased from the Vichi Industry (Pingtung, Taiwan). Sugar, salt, monosodium glutamate, five-spice powder, white pepper, rice wine, and sorghum wine were supplied by Yu-Hsuan Food Co. Ltd. (Pingtung, Taiwan). This study also used sodium nitrite and potassium nitrate (Yih-Yuan Food Co. Ltd., Keelung City, Taiwan), sodium phosphate (Emperor Chemical Co., Ltd., Hangzhou, China), and sodium erythorbate (Shun-Ching Ltd., Hong Kong, China). Nitrite added in sausages can help retain a bright red color and prevent botulism poisoning, ensuring food safety and preserving sensory characteristics and overall food quality. Nitrates are ubiquitous in the environment as a part of the natural nitrogen cycle, but their high concentrations in foods could be considered a health concern (e.g., due to the possibility of N-nitroso compounds formation) [8]. In this sense, the standards dictate that the calculated residual NO_2_ in meat products should not exceed a certain level. At the same time, many countries mandate the addition of nitrites or nitrates during sausage production to mitigate the botulinum poisoning risk. It also helps with maintaining meat color. Additionally, some research suggested that dietary nitrates and nitrites could pose cardiovascular benefits that may outweigh the risks [22]. Accordingly, this study considered nitrite as a variable in product formulation in the range below the maximum allowable doses [23].

### 2.2. Sample Preparation

#### 2.2.1. Salted Casing Processing

Hog casings were thoroughly rinsed to remove excess salt and soaked in 35 °C water for 20 min. Subsequently, the interior of the casings was cleaned properly and rinsed thoroughly in running water for one hour.

#### 2.2.2. Djulis Processing

Djulis grains were placed on a fine mesh sieve and then gently submerged in clean water to rinse off fine sand particles and saponins. The grains were steam-cooked for 15 min and then cooled to room temperature before use.

#### 2.2.3. Djulis-Based Sausage Processing

Djulis sausages were produced based on the traditional methodology of Taiwan’s indigenous people. First, fascia and membranes from the visible connective tissue on the raw meat were carefully removed. The lean meat was then ground through a 9.5 mm plate and formulated with the addition of non-meat ingredients. The ingredients used in this experiment consisted of brown sugar, monosodium glutamate, salt, five-spice powder, white pepper powder, rice wine, sorghum wine, polyphosphate, and sodium erythorbate. Different shell ratios of Djulis were evenly mixed with extra fat and lean meat ratios (0/100, 30/70, 50/50%) and different nitrite concentrations (0.07, 0.05, 0.03 g/kg). The original sausage sample (as the control treatment) was formulated with 70% pork lean meat, 30% backfat, and an additional 0.03% nitrite. Set quantities of phosphate and salt were added and stirred with a mixer for 10 min. The meat mixture was refrigerated for eight hours to be marinated before being stuffed into natural hog casings. The sausages were cured in a sausage dryer at 65 °C for 3 h. The links were then cooked for 90 min at 75 °C after vacuum packaging [24].

### 2.3. Experimental Design

#### 2.3.1. Questionnaire on Djulis Sausage Ingredients

Ten individual sausage producers were surveyed via a questionnaire to determine factors of control and standards. Nine variations of Djulis sausage Taguchi orthogonal arrays were selected, and after a confirmatory test, Taguchi analysis was used to identify the most suitable factors. Samples were analyzed through instrumental and sensory methodologies.

#### 2.3.2. Taguchi Method

The in-triplicate L_9_(3^4^) orthogonal array Taguchi method employed the information in Section 2.3.1 to evaluate the effects of four independent parameters, each at three levels, in nine Djulis sausage treatments, according to the Taguchi methodology recommended in the literature [16,25]. The independent parameters (control factors) were the un-hulled-to-hulled ratio of Djulis (A), pork backfat-to-lean meat ratio (B), low cooking temperature (C), and nitrite content (D). The factors and levels (ratio of sausage formula) of orthogonal array L_9_(3^4^) are shown in Table 2.

#### 2.3.3. Calculation of Signal-to-Noise Ratio (S/N)

After conducting the experiments, the Taguchi method uses a statistical performance measure as S/N values to establish the design with the highest quality and minimum variation. The quality characteristics of the S/N value were calculated based on three aspects, including “nominal-the-best (NTB),” i.e., the closer its quality characteristics are to the target, the better (Equation (1)), “smaller-the-best (STB),” i.e., the smaller the quality target, the better (Equation (2)), and “larger-the-best (LTB),” i.e., the larger the quality target, the better (Equation (3)).

(1) $$NTB=-10\times \log\frac{y^{z}}{s^{z}},\;f_{x}=10\times \log\langle {\left\{\left[\left(y_{1}+y_{2}+y_{3}\right)\right]/3\right\}}^{2}\rangle /{SD}^{2}$$

(2) STB=−10×log⁡∑inyi21n, fx=−10×log⁡(y12+y22+y323)

(3) LTB=(−10×log⁡∑in1yi12n), fx=−10×log⁡(1y1)2+(1y2)2+(1y3)2)/3

### 2.4. Sensory Evaluation Analysis

Sensory analysis was performed according to the method described in reference [26] with minor modifications. Cooked Djulis sausages were served to 120 untrained participants (aged 15–65) to evaluate the sensory attributes for appearance, aroma, bitterness, juiciness, and overall acceptability on a 7-point hedonic scale. Each item was scored between 1 and 7 (1: dislike extremely, 2: dislike moderately, 3: dislike slightly, 4: neither like nor dislike, 5: like slightly, 6: like moderately, 7: like extremely), except for bitterness which was scored between 7 and 1 (7: dislike extremely, 6: dislike moderately, 5: dislike slightly, 4: neither like nor dislike, 3: like slightly, 2: like moderately, 1: like extremely). At the same time, each panel member could not score items more than once. A 15 s interval was considered between each evaluation step. During each evaluation, the appearance was first observed and scored. Subsequently, the scoring was performed individually for aroma, bitterness, juiciness, and overall acceptability.

### 2.5. Physical Properties

#### 2.5.1. Texture Profile Analysis (TPA)

Samples, with dimensions of 9 mm diameter and 2 mm height were taken. The samples underwent a compression test using a Universal Testing Machine (model 5564, Instron Co., Columbus, OH, USA), and were analyzed using Instron 5.4 1.00 software. A 35 mm diameter probe was used with a compression ratio of 75%, and the compression speed of the probe was set at 60 mm/min. Each sample was compressed twice to achieve the force-time curve. Then, hardness, springiness, gumminess, and cohesion force were obtained [27,28].

#### 2.5.2. CIE Color Analysis

CIE values, including lightness (L*), redness-greenness (a*), and yellowness-blueness (b*) of the cross-sectional slices of sausage samples, were measured using a colorimeter (CR-310, Konica Minolta, Osaka, Japan) at room temperature [29].

### 2.6. Chemical Properties

#### 2.6.1. Peroxide Value Test

The POV test utilizes the redox potential in chemical reactions to measure the degree of oxidation of oils and fats. It involves the oxidation of iodide ions to iodine molecules by hydroperoxide, followed by a redox reaction between iodine molecules and sodium thiosulfate. The peroxide value (POV) is expressed as the milliequivalent number of peroxides per 1 kg of oil. POV is instrumental in assessing the initial oxidation of oils and fats, which is crucial for evaluating the deterioration of frying oil due to oxygen-induced oxidation. Subsequent polymerization reduces the peroxide value [30].

POV analysis was performed using the literature methodology [31] with minor modifications. Briefly, samples were dried at low temperatures and oil was extracted through the solvent extraction method at a controlled temperature. Then, 5 g of the extract was placed in a 250 mL triangular flask with a glass cover, mixed with 50 mL of acetic acid-isooctane solution containing 30 mL of glacial acetic acid with purity 99.8% (Honeywell, Offenbach, Germany) and 20 mL of isooctane with purity of 99.5% (Honeywell). The sample and the test solution were thoroughly dissolved and mixed by shaking. Afterward, 0.5 mL of saturated potassium iodide solution with purity of 99% (Honeywell) was added, shaken for 1 min, and mixed with 30 mL of distilled water. Finally, 0.01 N sodium thiosulfate solution with purity of 99%, (Honeywell) was used to titrate the sample until a yellow color appeared. Then, 0.5 mL of 10% SDS solution and 0.5 mL of 0.5% starch indicator were added. Titration with sodium thiosulfate solution continued until the blue precipitate disappeared, and the volume of sodium thiosulfate solution consumed was recorded to calculate the POV according to Equation (4).

(4) $$POV=\frac{\left(S-B\right){Na}_{2}S_{2}O_{3}mL\times 0.01\times F\cdot {Na}_{2}S_{2}O_{3}}{W}\times 1000$$

where: F is the normality of the sodium thiosulfate solution, S represents the sample group, B represents the blank group, Na_2_S_2_O_3_ mL is the volume of sodium thiosulfate used (m), and W is the sample weight (g).

#### 2.6.2. DPPH Test

The inhibitory effect against 2,2-Diphenyl-1-picrylhydrazyl (DPPH) is a common method to calculate the antioxidant capacity [32]. DPPH tests were performed for Djulis using a similar methodology reported in a previous study [14] with minor modifications. Ethanol was used as the solvent, and samples were mixed with 100 μM DPPH-ethanol solution at a ratio of 1:1. Then, the reaction was performed in dark conditions for 30 min. Finally, the samples were analyzed at the 517 nm wavelength within ELISA plates (Jet Bio-Filtration Co., Ltd., Guangzhou, China) using a microplate spectrophotometer (EP0CH, Biotech Epoch Microplate Spectrophotometer, Agilent, Santa Clara, CA, USA). DPPH free radical scavenging activity inhibition was then calculated and reported as a percentage.

### 2.7. Statistical Analysis

The data collected were statistically analyzed in triplicate using SPSS Statistics V.20.0. All data were subjected to analysis of variance (ANOVA) to assess the significance of the effects of treatments on Djulis sausages. Differences among the treatments’ means were considered significant at *p* < 0.05 using the Duncan multiple range test.

## 3. Results and Discussions

### 3.1. Appearance of Djulis Sausage

**Figure 1:**
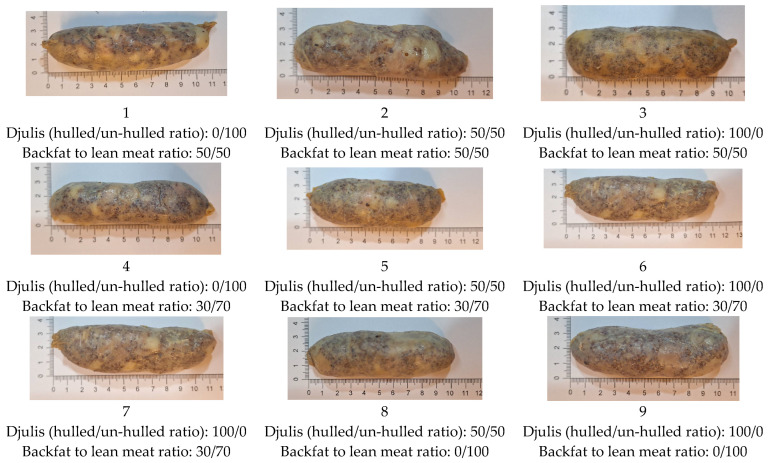
The appearance of nine batches of Djulis sausages made with different formulas. Numbers 1–9 under each picture refer to the treatment used according to the Taguchi method: 1: lean meat 50% and un-hulled Djulis 100%; 2: lean meat 50% and 50–50% of un-hulled to hulled Djulis; 3: lean meat 50% and hulled Djulis 100%; 4: lean meat 70% and un-hulled Djulis 100%; 5: lean meat 70% and 50–50% of un-hulled to hulled Djulis; 6: lean meat 70% and hulled Djulis 100%; 7: lean meat 70% and un-hulled Djulis 100%; 8: lean meat 100% and 50–50% of un-hulled to hulled Djulis; 9: lean meat 100% and hulled Djulis 100%.

The resulting Djulis sausages from nine treatments/formulas are shown in Figure 1. According to the results, as the proportion of lean meat increases, the visual color shifts towards a darker red. On the other hand, a higher proportion of hulled Djulis imparts a redder hue to the sausages due to the pigments primarily concentrated in the skin of Djulis. Thus, the visual color tends to become slightly lighter when Djulis is used as an ingredient in sausages. Overall, the appearance of the Djulis-enriched sausages was similar to that of traditionally produced sausages in the Southern region of Taiwan.

### 3.2. Sensory Evaluation

Results of the sensory evaluation by 120 participants on the nine treatments of Djulis sausages are shown in Table 3. The mean scores for appearance are sorted in descending order as follows: treatment 8 (6.56) > treatment 5 (6.17) > treatment 2 (5.90) > treatment 9 (5.46) > treatment 6 (5.29) > treatment 3 (5.14) > treatment 7 (3.67) > treatment 4 (3.57) > treatment 1 (3.37). Except for sausages in treatment 1, the appearance scores of treatments 2 to 9 all fall between very bright (7) and neither like nor dislike (4). The mean scores for aroma are sorted in descending order as follows: treatment 8 (6.40) > treatment 5 (5.94) > treatment 9 (5.78) > treatment 2 (5.54) > treatment 6 (5.23) > treatment 3 (5.04) > treatment 7 (3.99) > treatment 4 (3.78) > treatment 1 (3.53). The aroma scores of all treatments (1 to 9) fall between very strong (7) and neither like nor dislike (4).

The results represent the average value of three replications (*n* = 3) followed by the standard deviation (SD), i.e., mean ± SD.

The above-reported results were analyzed using the Taguchi method to clarify the effects of independent parameters on the sensory properties of samples, as discussed in Section 3.3.

The mean scores for bitterness are sorted in descending order as follows: treatment 8 (6.32) > treatment 2 (5.82) > treatment 5 (5.70) > treatment 9 (5.64) > treatment 6 (5.51) > treatment 3 (5.43) > treatment 7 (5.13) > treatment 4 (4.89) > treatment 1 (4.87). The bitterness scores of all treatments (1 to 9) fall between very non-bitter (7) and neither like nor dislike (4). The mean scores for juiciness are sorted in descending order as follows: treatment 9 (5.89) > treatment 8 (5.74) > treatment 6 (5.58) > treatment 5 (5.43) > treatment 2 (5.42) > treatment 3 (5.37) > treatment 7 (3.30) > treatment 4 (3.18) > treatment 1 (3.02). Except for sausages in treatment 1, treatment 4, and treatment 7, the appearance scores of treatments 2, 3, 5, 6, 8, and 9 fall between very juicy (7) and neither like nor dislike (4). The mean scores for overall acceptance are sorted in descending order as follows: treatment 8 (6.77) > treatment 5 (6.28) > treatment 2 (6.02) > treatment 9 (5.59) > treatment 6 (5.01) > treatment 3 (4.94) > treatment 7 (3.88) > treatment 4 (3.77) > treatment 1 (3.46). The overall acceptance scores of all treatments (1 to 9) fall between very, like extremely (7), and neither like nor dislike (4).

### 3.3. Taguchi Analysis of Sensory Aspects and Overall Acceptance of Djulis Sausage

Based on the sensory evaluation results, satisfaction with the overall acceptance characteristic of Djulis sausage was analyzed by the larger-the-best characteristic analysis (Table 4).

This study employed the Taguchi orthogonal to determine the influential parameters and levels for formulating Djulis sausage recipes. Nine groups of Djulis sausages were then produced and subjected to sensory evaluation. The sensory data characteristics were then analyzed for larger-the-better acceptance regarding the juiciness satisfaction of Djulis sausage products, as shown in Table 5. Results confirmed that the pork fat-to-lean ratio is the most critical factor affecting mouthfeel, followed by the Djulis ratio and cooking temperature. The least influential parameter was the nitrite content.

**Figure 2:**
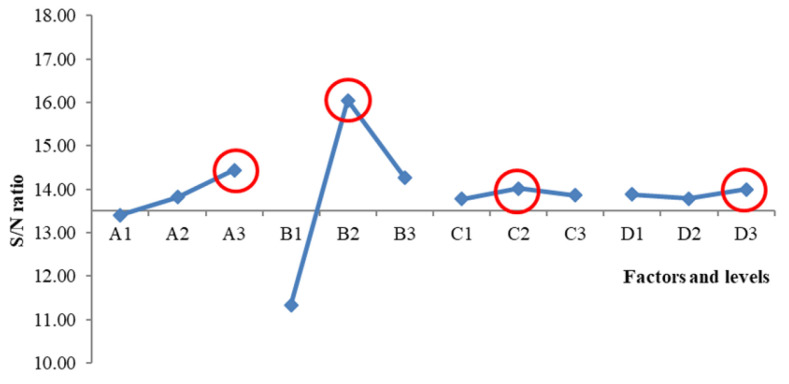
Signal-to-Noise (S/N) ratio response graph for the overall acceptance of Djulis sausage. The red circles indicate the highest S/N for each variable.

Moreover, the Taguchi S/N ratio response graph is shown in Figure 2. The highest plot of the S/N ratio suggested the most substantial effect. Hence, the analysis indicated that the best overall acceptability was obtained at an optimal parameter of A3 (16.8% of un-hulled Djulis), B2 (30/70 pork backfat-to-lean meat ratio), C2 (75 °C cooking temperature), and D3 (0.03 g/kg nitrite content).

The variance analysis of the overall acceptance is shown in Table 6. All four factors significantly impacted the overall acceptance of Djulis sausages. Among them, the pork backfat-to-lean meat ratio was the most influencing factor, followed by Djulis. The interactions between independent parameters are presented in the Supplementary Materials (Table S1 and Figure S1).

### 3.4. Verification Analysis of Results

Results of larger-the-best characteristic analysis for overall acceptance indicated that A3 (16.8% of un-hulled Djulis), B2 (30/70 pork backfat-to-lean meat ratio), C2 (75 °C low-temperature cooking), and D3 (0.03 g/kg nitrite content) are the best parameters for the optimum overall acceptability. Therefore, these results can be confirmed as the optimum formula for Djulis sausage. Its overall acceptance score falls between 7 (like extremely) and 6 (like moderately) with an S/N value of 16.63, which falls within the range (16.62~17.06) of the in-triplicate verification experiments (confidence interval below 95%) as shown in Table 7. Therefore, the strategy and effect of the Taguchi method can indeed discover an optimum formula to develop a novel Djulis sausage with high consumer acceptability.

Under the A_3_B_2_C_2_D_3_ optimization conditions, this verification experiment was performed three times, and 10 evaluators obtained a total of 30 data points (Y1 = 6.80, Y2 = 6.75, Y3 = 6.80). The average value of this verification experiment was 6.78 ± 0.03, and the S/N ratio was 16.63. The verification gain, calculated as (16.63 − 16.61)/16.61, amounted to 0.12%. This close correspondence between the optimization and experimental results, along with the minimal verification gain, underscores the reproducibility of the optimal conditions, affirming the robustness of the experimental outcomes.

### 3.5. Physical Properties of the Products

#### 3.5.1. Texture Profile Analysis of Sausages

**Figure 3:**
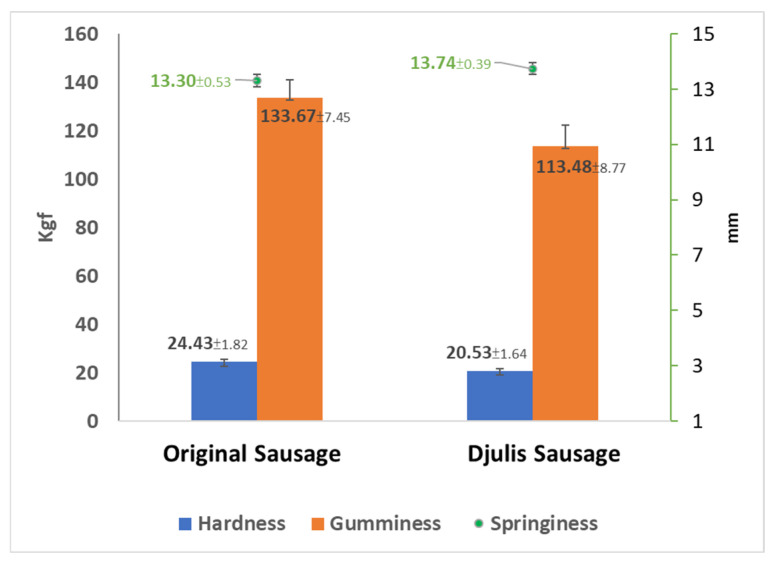
Selected values from texture profile analysis of Djulis sausages at the optimized formulation compared to the original sausage. Error bars indicate standard deviation. [Unit of measurement: hardness (Kgf), gumminess (Kgf), springiness (mm)].

The results of TPA for samples are presented in Figure 3. The original sausages had similar hardness and springiness values but higher gumminess than optimally formulated Djulis sausage. This finding differs somewhat from the study that compared Vienna sausages made solely from pork and those made with varying proportions of black soldier fly larvae and reported that formulation affects the hardness and cohesiveness values [33]. Therefore, low-temperature sous-vide cooking for Djulis sausages could be considered for novel food development.

At the same time, no significant differences were observed in hardness, springiness, and cohesion force (*p* > 0.05). Sausage textural properties could be influenced by meat quality and formulation. The results indicate that the gumminess between the tissues of the original sausages and Djulis sausages was not affected under both high-temperature steaming and low-temperature sous-vide cooking conditions.

Sous-vide cooking is a method that involves relatively lower temperatures and longer cooking times. The heating conditions typically fall between the critical points of denaturation for muscle proteins and collagen tissues. Different heating conditions may cause the denaturation of muscle proteins, resulting in tougher chicken meat or the dissolution of collagen tissues, leading to tender meat [34]. Meat hardness and tenderness are closely related to the heating conditions, especially in the temperature range of 60–70 °C, which is associated with the denaturation or hydrolysis of collagen proteins [35]. On the other hand, sous-vide cooking positively impacts the texture parameters [36]. Nevertheless, based on the data obtained in this study, although the sous-vide cooking technique resulted in some noticeable changes in gumminess, these variations could still be considered an acceptable texture range when referring to the sensory evaluation data discussed in the previous sections of this manuscript.

#### 3.5.2. CIE Color Values of Sausages

**Figure 4:**
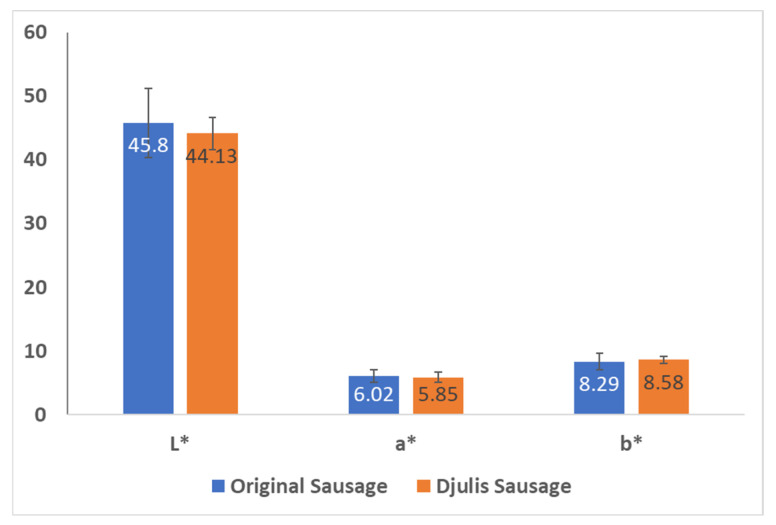
Analysis of sausage CIE color values. The results represent the average value of three replications (*n* = 3) as mean ± SD. L* is lightness. a* is redness and b* is yellowness; all values reported in this figure are dimensionless.

The color values (L*, a*, b*) are presented in Figure 4. It can be observed that the changes in the formulation did not significantly affect the CIE values of the samples after cooking. Therefore, enriching sausage with nutritionally valuable Djulis could enhance the overall nutritional values without compromising the product appearance, which is an important parameter in marketing. It has been previously explained that sous-vide could be considered a beneficial cooking method to achieve desirable products with minimum undesirable changes in color [37]. This finding is consistent with a previous study on the effect of sous-vide conditions on meat quality [38].

Considering similar properties of the innovative product developed in this study to those of the original sausage, the findings could be considered a step toward achieving healthier alternatives to so-called “possibly linked to health problems”, providing the industry with solutions to address this concern [39].

### 3.6. Chemical Properties of the Sausage Samples

#### 3.6.1. Peroxide Value Test

**Figure 5:**
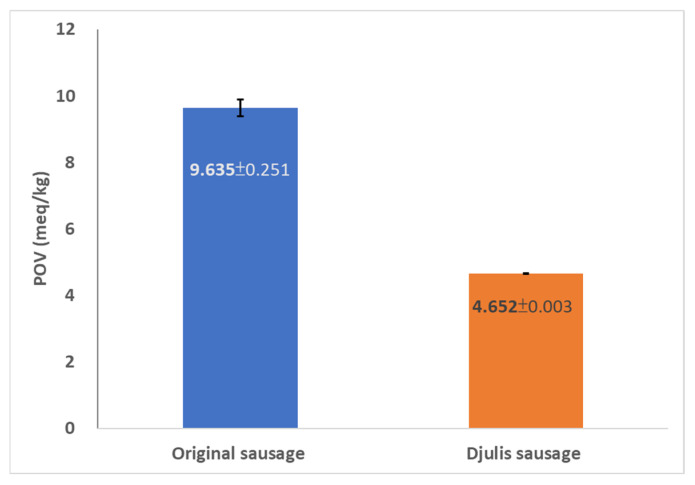
The result of the peroxide value test of sausage samples. Data represented as mean ± SD.

This report is among the first studies to analyze the effect of Djulis on the oxidative stability of meat products. Results showed that incorporating Djulis in the sausage at the Taguchi-defined optimized formulation could significantly enhance the sausage oxidation stability and reduce the POV by 51.7% (Figure 5). While limited information has been documented on the preservative effect of red quinoa, a recently conducted study demonstrated a decrease in POV from 3.5 meq/kg to 1.0 meq/kg by incorporating 0.4 g/kg seeds of Chenopodium quinoa in Japanese quail [40]. It has been reported that lipid oxidation in meat products could reduce the sensory properties and negatively affect the consumers’ health [41]. Therefore, novel product formulations that could hinder the oxidation provide benefits to the meat industry.

In our study, the optimized sausage product comprised 100% unhulled Djulis, a pork fat-to-lean ratio of 30/70 (90 g/210 g), a low cooking temperature of 75 °C, and a nitrite content of 0.03 mg/g. A comparative analysis with the control group, devoid of Djulis, revealed a POV of 4.652 meq/kg (with a standard deviation of 0.003) for the experimental group. This value was significantly lower than the 9.635 meq/kg (with a standard deviation of 0.251) recorded for the control group, suggesting that Djulis can effectively mitigate meat spoilage by influencing the peroxide value. This observation contributes valuable insights into the potential preservative properties of Djulis in meat products.

#### 3.6.2. DPPH Test

Results showed that Djulis used in the present study has a significant antioxidant activity with a DPPH inhibitory rate of 62.368%. This is the reason behind the observation discussed in Section 3.6.1 of this manuscript, suggesting the enhanced oxidation stability of sausage (reduced POV) could be due to the presence of bioactive compounds of Djulis with free radical scavenging activity antioxidant properties. Previous studies also indicated a significant antioxidant effect for quinoa, which is believed to be correlated with its hydrophilic (e.g., phenolic compounds) and hydrophobic qualities, such as carotenoids and tocopherols [42]. In a recent study, the DPPH value was increased from 20.3 to 34.5% when 12.5% quinoa seed was incorporated in a chicken meatball formulation [43]. Other researchers also suggested that enhanced quality of meat products during storage could be linked to antioxidant effects, such as considerable DPPH inhibitory values, for products formulated with bioactive-rich plants [44].

The antioxidant activity of Djulis, as an ingredient in Djulis-enrich sausage, is linked to its chemical compositions. A Djulis sample, originated from the same region as one used in present study, has been comprehensively analyzed by experts using high-performance liquid chromatography-diode array detection-tandem mass spectrometry method (HPLC-DAD-MS/MS) [45]. The study utilized several standard compounds, followed by method validation, identifying fourteen flavonoids and eight phenolic acids in the Djulis sample. Hydroxyphenylacetic acid pentoside, with a concentration of 1855.26 μg/g, was the major phenolic acid. The sample was also rich in other phenolic compounds such as vanillic acid, hydroxyphenylacetic acid, vanillic acid hexoside, quinic acid, caffeoyl-putrescine-derivative, caffeoyl-spermine-conjugate and caffeoyl-putrescine-derivative with concentrations of 70.66, 55.42, 45.03, 37.00, 31.70, 13.11, and 11.50 μg/g, respectively. They also reported that the major flavonoids was rutin-O-pentoside (257.30 μg/g), followed by 72.78, 70.69, 67.57, 57.29, 44.37, 35.92, 35.19, 30.36, 28.14, 25.15, 23.80, 22.25, and 19.93 μg/g quercetin-acetyl-rutinoside hexoside glucuronide, quercetin-3-O-(coumaroyl)-rutinoside (2), rutin-O-pentoside, quercetin-acetyl-rutinoside, quercetin-acetyl-rutinoside, rutin (quercetin-3-O-rutinoside), quercetin-3-O-(coumaroyl)-rutinoside deoxyhexoside, quercetin-3-O-(coumaroyl)-rutinoside, quercetin-acetyl-rutinoside hexoside, quercetin-3-O-(coumaroyl)-rutinoside pentoside, quercetin-acetyl-rutinoside hexoside, quercetin-acetyl-glycoside, and quercetin-3-O-(coumaroyl)-rutinoside pentoside, respectively. It has been previously well-explained how the chemical structures of phenolic acids and flavonoids correlate with their antioxidant activity [46]. In another study, researchers analyzed the bioactive profile of the hull of Djulis collected from the southern part of Taiwan (the same as the Djulis collection area in the current study). They found Djulis hull rich in flavonoids (~57 mg QU/g) and phenolics (~642 mg GAE/g) [47]. They also identified rutin, vitexin, and naringin among primary flavonoids and gallic, chlorogenic, and coumaric acids as major phenolics. They also explained that various parameters, such as extraction methods and conditions, could affect the concentrations of these compounds. Accordingly, Taguchi-optimized Djulis-enriched sausage could contain significant amounts of bioactive components, such as the phenolics and flavonoids listed above, with some possible variations, which could be the reason behind its improved oxidation stability.

## 4. Conclusions

Sensory evaluation results showed that formulation could significantly affect the acceptability of Djulis-enriched Chinese-style sausage. Taguchi was successfully used as an optimizing methodology to formulate a novel product while considering multiple variants and reducing the number of experiments. Overall acceptance analysis suggested the optimized formulation with the desirable S/N is A_3_B_2_C_2_D_3_, i.e., 16.8% of un-hulled Djulis, 30/70 backfat-to-lean meat ratio, 75 °C cooking temperature, and 0.03 g/kg nitrite. While all independent parameters significantly impacted the overall acceptance, the pork backfat-to-lean meat ratio was the most impactful variable, followed by Djulis incorporation. Additionally, the optimized Djulis-enriched sausage recipe was verified with an overall acceptance of 6–7 (like extremely-to-like moderately), suggesting its potential marketability. Djulis-enriched sausage at the optimal formulation had a reduced fat content, improved oxidation stability, and enhanced bioactive content. These could provide the industry with an alternative meat product for future market expansion.

## Figures and Tables

**Table 2:** Factors and levels for the L_9_(3^4^) orthogonal design.

Factor/Level	Independent Parameters	1	2	3
A	Djulis (hulled:un-hulled %)	100:0	50:50	0:100
B	Backfat to lean meat ratio (%)	0/100	30/70	50/50
C	Cooking temperature (°C)	55	75	95
D	Nitrite (g/kg)	0.07	0.05	0.03

**Table 3:** Taguchi design matrix depicted as recipe * of Djulis-enriched sausage.

Treatments/ Main Ingredients (%)	Hulled Djulis	Un-Hulled Djulis	Pork Lean	Pork Backfat	Nitrite
1	16.8 **	-	100	-	0.023
2	16.8	-	70	30	0.016
3	16.8	-	50	50	0.01
4	8.4	8.4	100	-	0.01
5	8.4	8.4	70	30	0.023
6	8.4	8.4	50	50	0.016
7	-	16.8	100	-	0.016
8	-	16.8	70	30	0.01
9	-	16.8	50	50	0.023

* The common ingredients were 6% sugar, 1% MSG, 1% salt, 0.012% five-spice, 0.3% white pepper, 5% rice wine, 0.2% polyphosphate, 0.05% sodium erythorbate, and 0.03% nitrite. ** % is calculated based on 300 g of lean meat, equivalent to 100%.

**Table 4:** Effect of nine treatments on the sensory evaluation of Djulis sausage.

No.	Appearance	Aroma	Bitterness	Juicy	Overall Acceptance
1	3.37 ± 0.24 ^c^	3.53 ± 0.15 ^c^	4.87 ± 0.20 ^b^	3.02 ± 0.25 ^c^	3.46 ± 0.22 ^c^
2	5.90 ± 0.20 ^a^	5.54 ± 0.07 ^a^	5.82 ± 0.08 ^a^	5.42 ± 0.10 ^b^	6.02 ± 0.13 ^a^
3	5.14 ± 0.16 ^b^	5.04 ± 0.10 ^b^	5.43 ± 0.24 ^b^	5.37 ± 0.22 ^b^	4.94 ± 0.11 ^b^
4	3.57 ± 0.22 ^c^	3.78 ± 0.08 ^c^	4.89 ± 0.15 ^b^	3.18 ± 0.15 ^c^	3.77 ± 0.12 ^b^
5	6.17 ± 0.15 ^a^	5.94 ± 0.05 ^a^	5.70 ± 0.12 ^a^	5.43 ± 0.09 ^b^	6.28 ± 0.13 ^a^
6	5.29 ± 0.14 ^b^	5.23 ± 0.06 ^b^	5.51 ± 0.17 ^b^	5.58 ± 0.05 ^a^	5.01 ± 0.05 ^b^
7	3.67 ± 0.09 ^c^	3.99 ± 0.14 ^c^	5.13 ± 0.06 ^b^	3.30 ± 0.12 ^c^	3.88 ± 0.13 ^b^
8	6.56 ± 0.08 ^a^	6.40 ± 0.12 ^a^	6.32 ± 0.19 ^a^	5.74 ± 0.08 ^a^	6.77 ± 0.06 ^a^
9	5.46 ± 0.04 ^b^	5.78 ± 0.15 ^a^	5.64 ± 0.16 ^b^	5.89 ± 0.15 ^a^	5.59 ± 0.15 ^a^

^a,b,c^ Means within the same column with different letters significantly differ at *p* < 0.05.

**Table 5:** Results of overall acceptance for all treatment combinations and their S/N ratios.

L_9_(3^4^)	Factor				Overall Acceptance Results			Ave	SD	S/N
	A	B	C	D	Y1	Y2	Y3			
EXP. 1	1	1	1	1	3.70	3.40	3.27	3.46	0.22	10.74
EXP. 2	1	2	2	2	6.17	5.93	5.97	6.02	0.13	15.59
EXP. 3	1	3	3	3	5.07	4.87	4.90	4.94	0.11	13.88
EXP. 4	2	1	2	3	3.87	3.80	3.63	3.77	0.12	11.51
EXP. 5	2	2	3	1	6.13	6.37	6.33	6.28	0.13	15.95
EXP. 6	2	3	1	2	5.07	4.97	5.00	5.01	0.05	14.00
EXP. 7	3	1	3	2	3.73	3.90	4.00	3.88	0.13	11.76
EXP. 8	3	2	1	3	6.80	6.80	6.70	6.77	0.06	16.61
EXP. 9	3	3	2	1	5.73	5.43	5.60	5.59	0.15	14.94
Ave								5.08	0.12	13.89

**Table 6:** Response of S/N ratio for the overall acceptance of Djulis sausage.

Level	Factor			
	A	B	C	D
1	13.40	11.34	13.78	13.88
2	13.82	16.05	14.01	13.78
3	14.44	14.27	13.86	14.00
Effect	1.03	4.71	0.23	0.22
Rank	2	1	3	4

**Table 7:** ANOVA results for the overall acceptance of Djulis sausage.

Factor	SS	DOF	Var	F Ratio	Confidence	Significant
A	1.69	2	0.84	49.46	100.00	***
B	31.88	2	15.94	933.23	100.00	***
C	0.04	2	0.02	1.13	65.51	*
D	0.17	2	0.09	5.02	98.15	**
Error	0.31	18	0.02			
Total	34.08	26				

*, **, and *** indicate significant differences at *p* < 0.05, <0.01, and <0.001, respectively.

## Data Availability

The data presented in this study are available on request from the corresponding author. The data are not publicly available due to data confidentiality purposes, each member of the panel was assigned a random three-digit code. Data were treated anonymously and following the European General Data Protection Regulation (Regulation E.C. (2016). No 679/2016 of the European Parliament and of the Council of 27 April 2016) and the Taiwan Personal Data Protection Act.

## Supplementary Materials

The following supporting information can be downloaded at: https://www.mdpi.com/article/10.3390/foods13010091/s1, Figure S1: Analysis of the interactions between the examined factors; Table S1: Correlation coefficients of independent parameters.
