# Supplementary material for: Development of Chinese-Style Sausage Enriched with Djulis (Chenopodium formosanum Koidz) Using Taguchi Method: Applying Modern Optimization to Indigenous People’s Traditional Food
Source: Foods. 2023 Dec 26;13(1):91. doi: 10.3390/foods13010091 (PMC10778872; doi:10.3390/foods13010091)
Supplement: Supplementary file 1 [file foods-13-00091-s001.zip › foods-2722948-supplementary.pdf]

Table S1. Correlation coefficients of independent parameters.

	A	B	C	D
A	1	0.525	0.237	0.634
B	0.525	1	.951	-0.325
C	0.237	0.951	1	-0.601
D	0.634	-0.325	-0.601	1

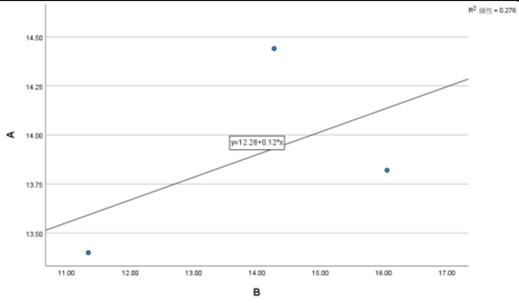

A-B correlation coefficient:0.525

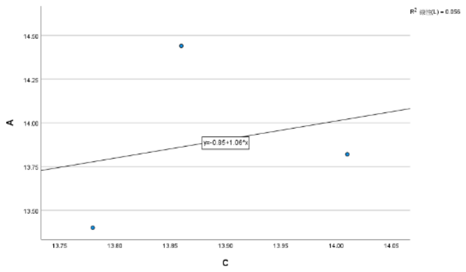

A-C correlation coefficient:0.237

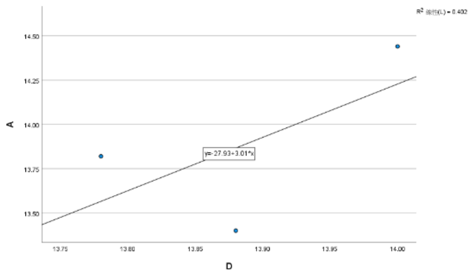

A-D correlation coefficient:0.634

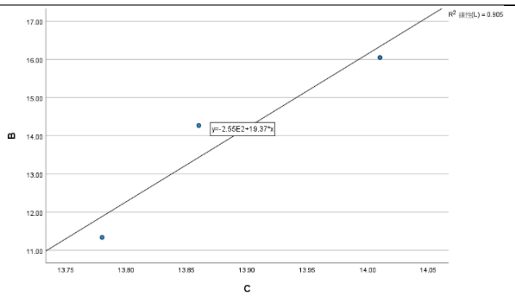

B-C correlation coefficient:0.951

Figure S1. Analysis of the interactions between the examined factors.
